# A Multifunctional Guide for Non‐Carious Cervical Lesion Restoration and Root Coverage Using a Coronally Advanced Flap in the Treatment of Combined Lesions

**DOI:** 10.1111/jerd.13485

**Published:** 2025-05-05

**Authors:** Isabella Neme Ribeiro dos Reis, Aldrin André Huamán‐Mendoza, Oswaldo Villa‐Campos, Nataly Zambrana, Newton Sesma, Franz Josef Strauss, Giuseppe Alexandre Romito

**Affiliations:** ^1^ Department of Stomatology, Division of Periodontics, School of Dentistry University of São Paulo São Paulo Brazil; ^2^ Department of Reconstructive Dentistry, Center of Dental Medicine University of Zurich Zurich Switzerland; ^3^ Department of Biologic and Material Sciences, School of Dentistry University of Michigan Ann Arbor Michigan USA; ^4^ Department of Prosthodontics, School of Dentistry University of São Paulo São Paulo Brazil; ^5^ Faculty of Health Sciences Universidad Autónoma de Chile Santiago Chile

**Keywords:** coronally advanced flap, digital workflow, gingival recession, non‐carious cervical lesion, surgical guide

## Abstract

**Objectives:**

This case report describes a digital workflow that integrates restorative and surgical planning through the design and fabrication of a multifunctional guide to optimize outcomes in the treatment of combined lesions.

**Clinical Considerations:**

Gingival recessions frequently occur alongside non‐carious cervical lesions (NCCLs), forming combined defects that complicate cementoenamel junction (CEJ) identification and treatment planning. A 40‐year‐old male presenting with gingival recession and NCCLs on teeth #14 and #15. A restoration was planned to reconstruct the lost CEJ using clinical reference points. A custom surgical guide was subsequently designed to replicate the planned restoration’s contour and to delineate the intended incision patterns for the coronally advanced flap (CAF) procedure. The CEJ restoration was executed using composite resin, with the guide assisting in achieving precise contouring. Root coverage was then performed using a CAF combined with a connective tissue graft, with the guide facilitating accurate scalpel blade placement during flap preparation. At the one‐year follow‐up, complete root coverage was observed and the restoration maintained stability in terms of marginal adaptation, color, and surface integrity.

**Conclusions:**

The use of a multifunctional digital guide can enhance the precision of both restorative and surgical procedures in the management of combined gingival recession and NCCL defects, potentially improving treatment predictability and outcomes.

**Clinical Significance:**

This case report highlights the benefits of a 3D‐printed multifunctional digital guide for precise CEJ restoration and flap design in treating gingival recessions associated with NCCLs.

## 1 Introduction

Gingival recessions are defined by the apical displacement of the gingival margin relative to the cementoenamel junction (CEJ), exposing the root surface [1]. They are highly prevalent worldwide, affecting individuals regardless of oral hygiene or periodontal status [2, 3, 4]. Treatment typically involves root coverage procedures such as coronally advanced flaps (CAFs), which may be performed with or without connective tissue grafts (CTGs), tissue substitutes, or biological agents [5, 6, 7].

Dental surfaces with gingival recession often also exhibit non‐carious cervical lesions (NCCLs), with approximately 50% of recession defects associated with NCCLs, forming a combined defect [8, 9]. NCCLs refer to the loss of tooth substance (crown, root, or both) at the cervical area, unrelated to dental caries. These lesions have a multifactorial etiology, with mechanical forces causing root abrasion as the primary etiological factor [10]. NCCLs complicate CEJ identification, which is critical for root coverage surgery and outcome assessment. Their presence worsens the prognosis [11], by increasing the likelihood of apical displacement of the gingival margin [12]. Additionally, root coverage may not completely cover the entire defect, making complete coverage more challenging [11, 13].

Effective management of these cases requires a tailored treatment strategy that considers both periodontal parameters and NCCL characteristics, including lesion depth and extent [9, 13]. A restorative approach is often needed for optimal outcomes and should be guided by a thorough clinical evaluation. When NCCLs compromise the CEJ reference, its restoration is recommended before root coverage [9, 13].

The apical limit of the restoration should be determined by the expected maximum coverage line, which corresponds to the predicted highest level the gingival margin may reach after root coverage [14, 15]. In cases of RT1 gingival recession, this estimate aligns with the CEJ position [14, 15]. However, even with careful planning based on anatomical references, errors may occur when defining the CEJ during restoration, potentially compromising the final outcome of root coverage [14, 15]. Additionally, flap design is a critical step in the procedure, and poor planning or execution can adversely affect surgical outcomes [16, 17].

With advances in digital technologies such as intraoral scanning, planning software, and both additive and subtractive manufacturing techniques, various surgical and restorative approaches in dentistry have been significantly improved in terms of planning and execution [18, 19, 20, 21, 22, 23]. Through digital planning on virtual models, it is possible to mill or print guides for procedures such as implant placement [23, 24, 25], harvesting of autogenous soft tissue grafts [26], sinus floor elevation [27], autogenous bone block retrieval [28], root coverage [29] and crown lengthening [30]. Moreover, guides can assist in direct restorative procedures [31, 32, 33].

To our knowledge, the use of guides for treating combined lesions, specifically the combination of gingival recessions and NCCLs, has not been reported before. Given the clinical challenges, guides assisting both restorative and surgical procedures could improve treatment predictability and outcomes. This case report describes a novel approach integrating digital planning and the fabrication of a multifunctional guide, aiding NCCL restoration and flap incisions for root coverage.

## 2 Case Report

This case report adheres to the CARE guidelines [34], with both oral and written consent obtained from the patient for treatment and the publication of this manuscript and accompanying images.

A 40‐year‐old male patient presented with combined lesions on teeth #14 and #15, gingival recession, and complaints related to esthetics and dentinal hypersensitivity. He reported a diet high in acidic foods and the use of a hard‐bristled toothbrush with excessive brushing force. Initially, contributing factors were addressed; the patient was instructed to adopt a gentler brushing technique using a soft‐bristled toothbrush, and dietary modifications were recommended to reduce acidic food consumption. Occlusal evaluation revealed balanced occlusion with adequate contacts in maximum intercuspation, lateral excursion, and protrusion.

A comprehensive periodontal examination confirmed periodontal health in a reduced periodontium [35]. Professional prophylaxis was performed, followed by intraoral photography and digital scanning. The dimensions of the NCCLs were clinically measured.

**FIGURE 1:**
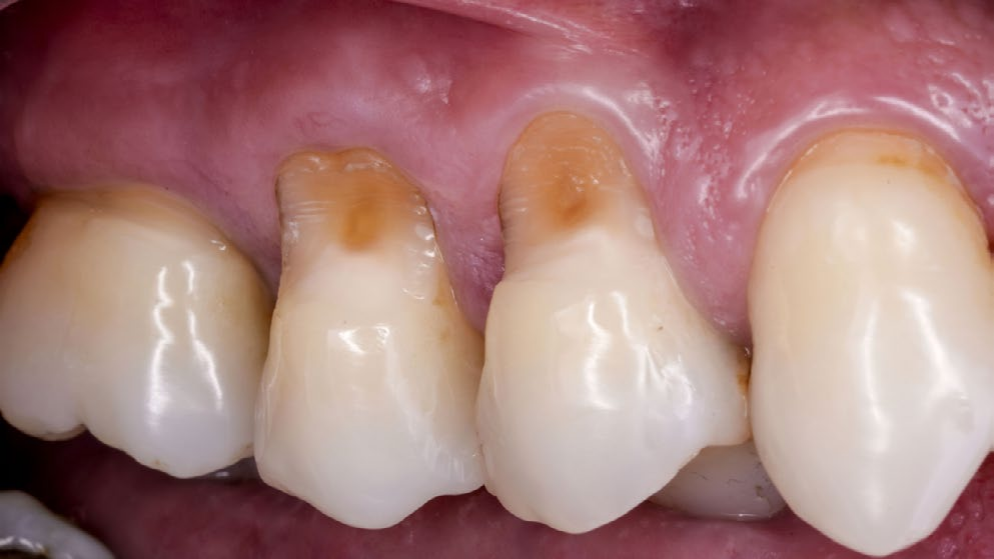
Initial aspect of the combined lesion (frontal view).

**FIGURE 2:**
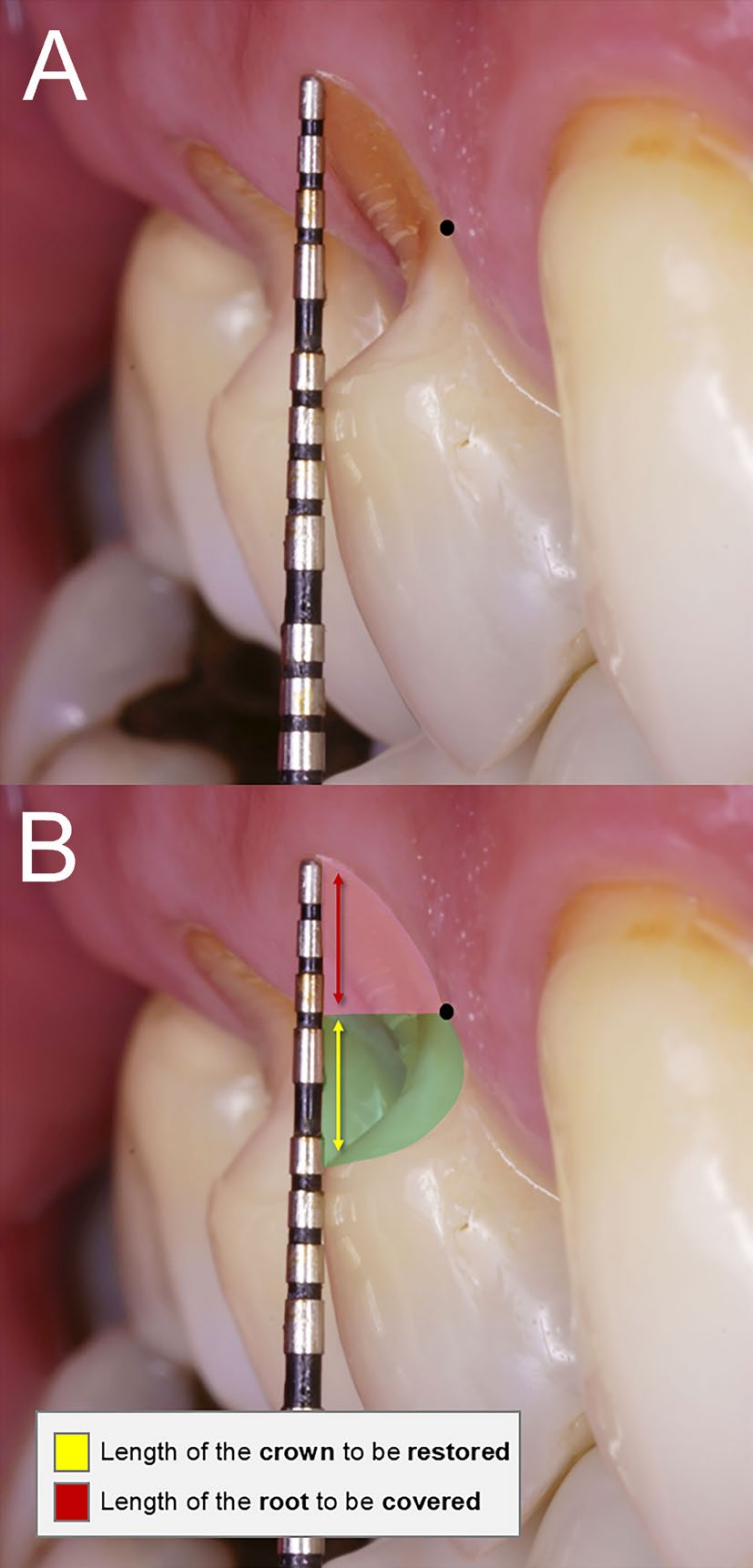
(A) Measurement of the combined lesion, approximately 6 mm in length on teeth #14 and #15. The black dot marks the point where the enamel ends and the radicular dentin begins, and the cementoenamel junction is visible in a lateral view. (B) On tooth #15, approximately 3 mm corresponds to the loss of coronal structure, and 3 mm corresponds to gingival recession.

- Tooth #14: Lesion length of 6 mm, measured from the coronal end of the defect to the gingival margin, and a depth of 3 mm.
- Tooth #15: Lesion length of 6 mm, with a depth of 2 mm (Figures 1 and 2).

Based on the Pini Prato classification [9], both lesions were categorized as Class B+, indicating deep NCCL affecting both the root and the coronal portion, with CEJ loss. A pronounced step‐like defect was noted along both the coronal and apical borders. No interproximal attachment loss was observed, and the gingival recessions were classified as RT1 [36, 37]. Additionally, a 2‐mm band of keratinized mucosa and a 1‐mm gingival thickness were noted in the affected area (Figure 1) [37].

### 2.1 Treatment Planning

For gingival recession associated with Class B+ NCCLs, it is recommended to restore the most coronal portion of the NCCL corresponding to the lost CEJ, followed by a mucogingival procedure for root coverage [9]. Adjacent teeth #13 and #16 presented minor conditions: tooth #13 exhibited a small recession of approximately 1 mm in length and tooth #16 had a minor NCCL, both asymptomatic and without associated complaints (Figure 1).

**FIGURE 3:**
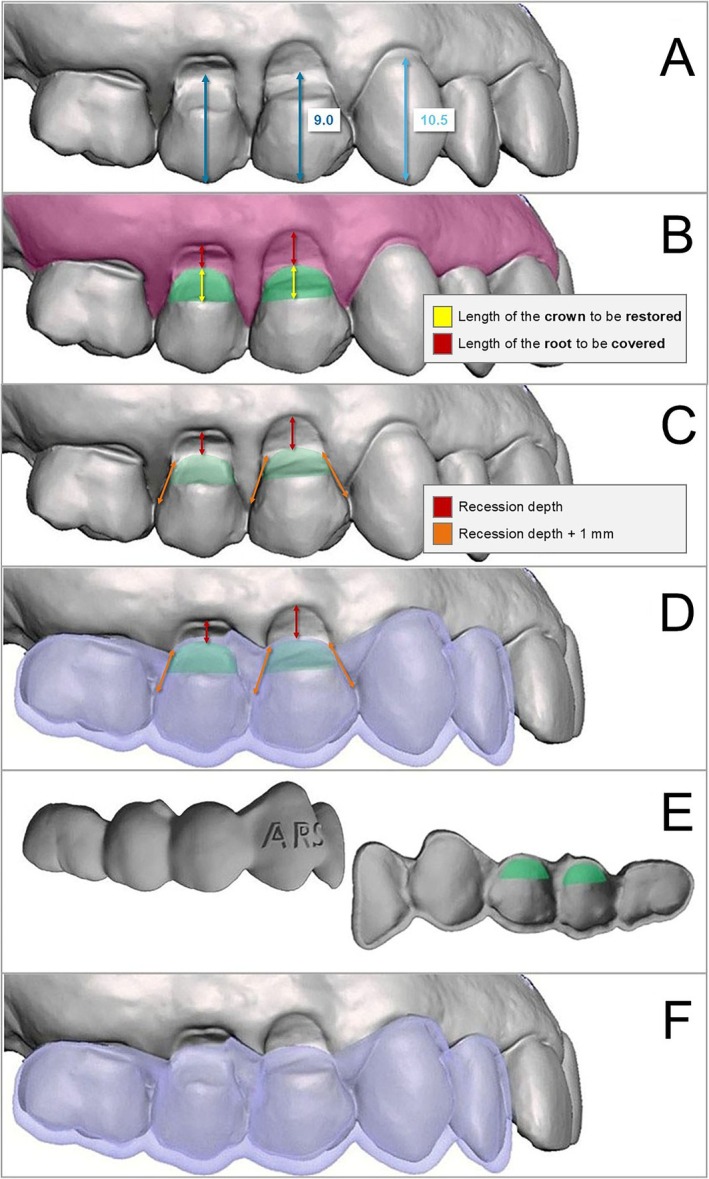
3D simulation and design of the guide. (A) Crown length measurements for teeth #14 and #15, based on the canine reference (10.5 mm), estimating the crown lengths for the premolars. (B) Yellow line represents the height of the area to be restored, and the red line indicates the height of the area to be covered. (C) Recession depth measurement (red line) and recession depth plus 1 mm (orange line) defining the incision pattern for CAF. (D) Surgical guide designed according to the previously mentioned parameters. (E) Frontal view of the guide showing incision limits, and internal view showing the restoration boundaries. (F) Frontal view of the guide.

Before surgical planning, the restoration of the CEJ was designed. Reference points included the visible end of the enamel and the beginning of the radicular dentin, which was clearly identifiable and used as a reference in both teeth (mesial and distal line angles, black dot in Figure 2) [13, 14]. The canine was also used as a reference point, since the canine had a 10.5 mm length and the ideal canine‐premolar proportion is 1.16 [38], the CEJ restoration was positioned approximately 3 mm from the gingival margin of tooth #14 and 2 mm from tooth #15, resulting in final tooth lengths of approximately 9 mm. The remaining 3 mm apically corresponded to the root structure, which would later be covered through surgery (Figure 3A,B).

Given the favorable anatomical conditions, a CAF was selected for multiple recessions, incorporating oblique incisions [39]. Due to thin gingival thickness and the presence of NCCLs [5, 40], a CTG was planned, using tooth #14 (with the greatest recession, 3 mm) as a reference. Recession length was measured, and 1 mm was added to this measurement. This total was then transposed along the mesial and distal margins of tooth #14, defining the endpoints of the oblique incisions. The oblique incision started at the adjacent teeth gingival margins and extended to the predetermined points [39]. The same procedure was subsequently applied to the distal aspect of tooth #15 (Figure 3C). For the mesial aspect of tooth #13, instead of incisions, a tunneling approach was chosen to enhance flap mobility while minimizing surgical trauma.

### 2.2 Guide Design and Fabrication

Autodesk Meshmixer software (version 3.5.474—Autodesk Inc. USA) was used to simulate the restoration, considering the established clinical parameters (Figure 3D). A custom surgical guide was designed based on the restored model. The internal portion of the guide followed the exact contour of the planned restoration, while its apical portion extended to match the apical limit of the restoration (Figure 3D,E). Additionally, the apical margin of the guide was designed to replicate the planned oblique incision pattern, specifically at the distal aspect of tooth #15 and both the mesial and distal aspects of tooth #14 (Figure 3D–F). This design was intended to facilitate precise scalpel blade placement while taking the restored CEJ into account during the upcoming surgery. The guide was then printed (Formlabs, United States).

**FIGURE 4:**
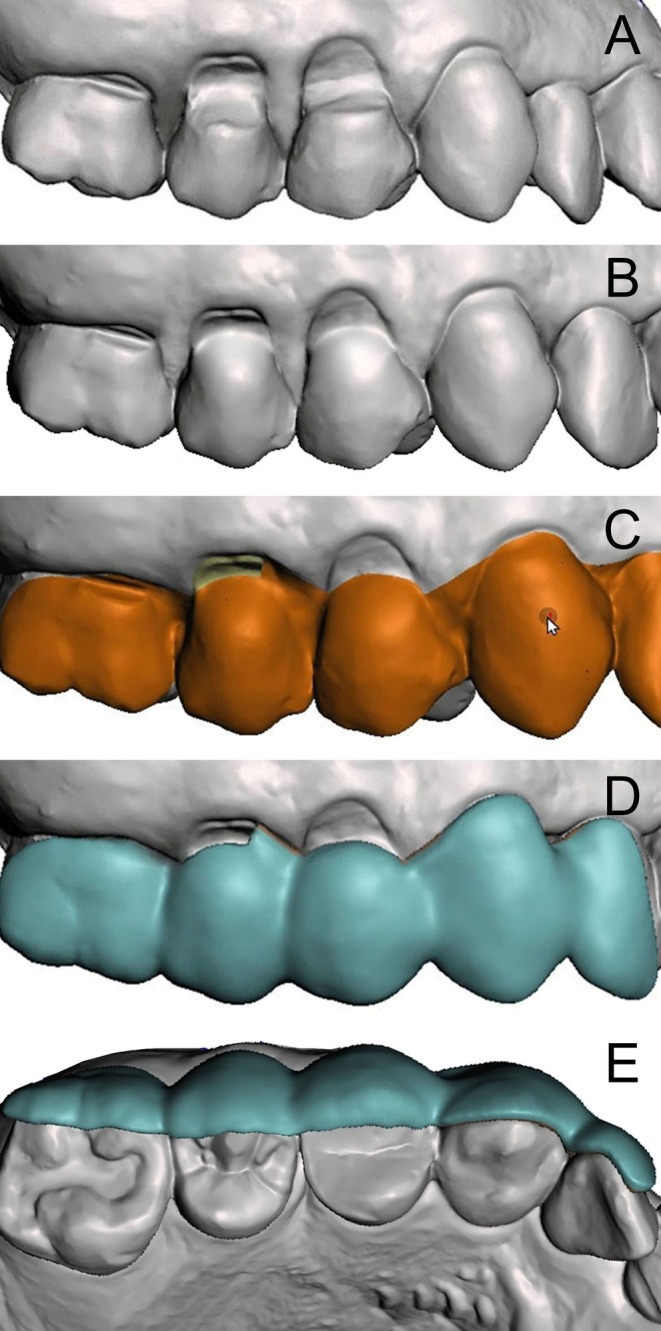
Guide design process.

The step‐by‐step design process was as follows:

1. Model preparation and guide insertion axis: The digital model of the upper jaw was opened in the software to model the surgical guide (Figure 4A). The insertion axis of the guide was identified from the buccal aspect, and based on this axis, reliefs were created in areas with excessive retention to ensure a proper fit of the guide. Using the “Sculpt” tool, reliefs were added, simulating wax application to prevent interference or difficulties during guide insertion while maintaining alignment with the insertion axis.
2. Restoration sculpting: The planned restorations for teeth #14 and #15 were sculpted using the same tool (Figure 4B), following clinical references from the clinical examination. The canine height was measured with a ruler, and the restorations were designed to extend 1.5 mm coronally, with contours shaped as previously described.
3. Mesh definition and guide design: The “Select” tool was used to define the mesh anatomy for generating the guide. Using the brush tool, mesh triangles corresponding to the guide area were selected, ensuring precise adaptation to the completed restorations and alignment with planned root coverage incisions (Figure 4C). The incisions were designed based on the restored teeth, setting the apical limits of the guide. Support was also defined on involved and adjacent teeth, incorporating portions of their incisal and occlusal surfaces.
4. Mesh duplication and final adjustments: After selection, the mesh was duplicated. The first duplication used the “Offset” tool to create a 0.10 mm fitting layer. A second duplication defined the thickness of the guide, which was adjusted to 1.5 mm, as required. Finally, the guide was refined, with edge adjustments and other necessary modifications to ensure a precise fit and optimal functionality (Figure 4D,E).

### 2.3 CEJ Restoration

**FIGURE 5:**
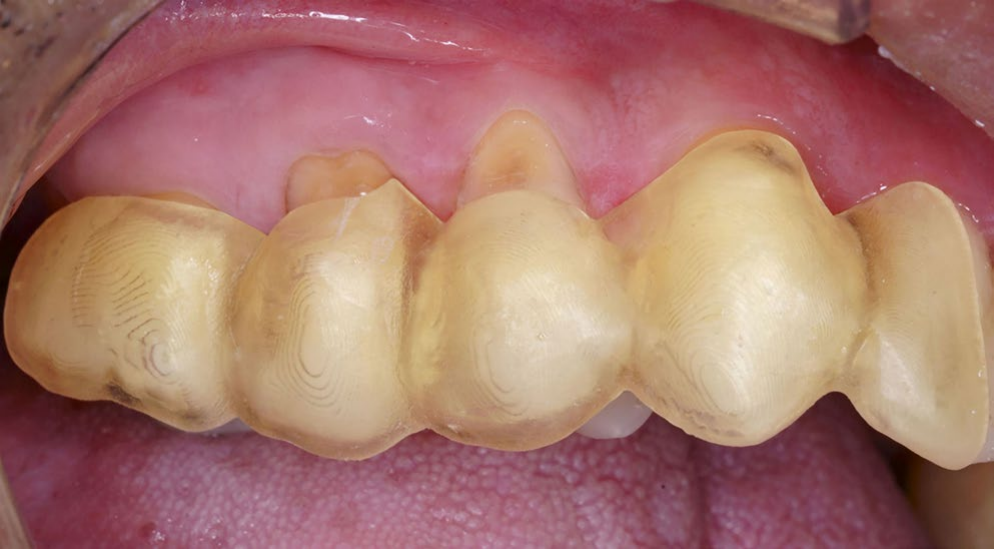
Guide adapted over the teeth.

**FIGURE 6:**
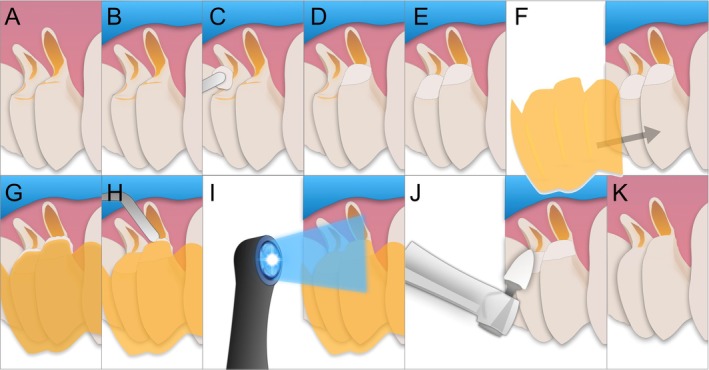
Restoration sequence using the multifunctional guide. (A) Initial aspect. (B) Modified rubber dam in place. (C) Composite resin applied to the cavity. (D) Composite resin placed on tooth #14. (E) Composite resin placed on tooth #15. (F) Guide positioned to ensure proper restoration contour. (G) Excess material. (H) Excess material removal. (I) Light‐curing completed with the guide in position. (J) Polishing. (K) Final result.

Before surgery, CEJ restoration was completed. Initially, the guide was tested for fit, demonstrating excellent adaptation (Figure 5). The complete sequence for performing the restorations using the multifunctional guide is shown in Figure 5. Isolation was achieved using a modified rubber dam technique (Figure 6A,B) [41], and the NCCLs were cleaned with a low‐speed rubber cup and pumice. Enamel was etched with 37% phosphoric acid (Biodinâmica, Brazil) for 30 s, and the dentin was treated with a self‐etching primer from a two‐step adhesive system (Clearfil SE Bond, Kuraray, Japan). The adhesive was applied according to the manufacturer's protocol.

**FIGURE 7:**
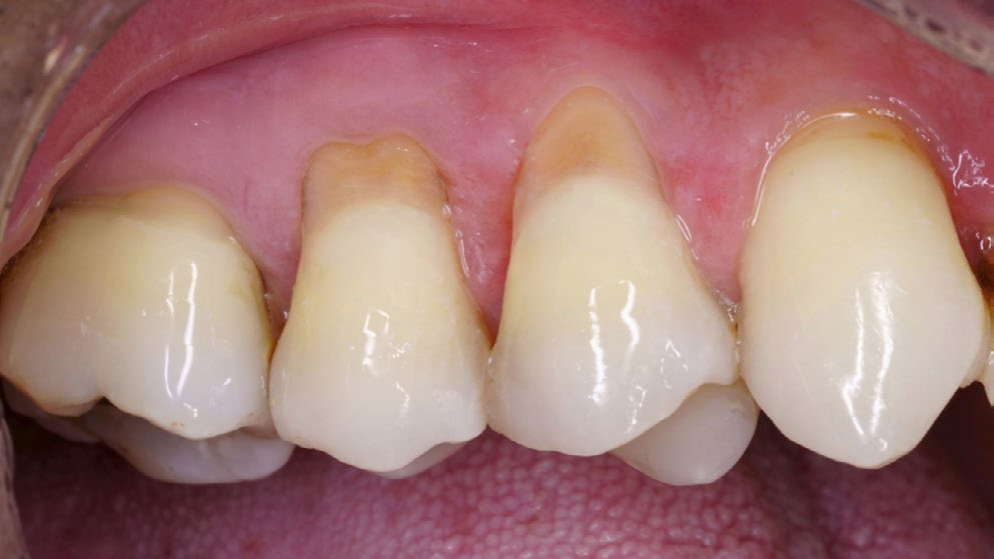
Composite resin restorations based on digital planning and printed guide, with apical CEJ limit and buccal volume guided by the guide. Photo taken after removing the modified rubber dam isolation.

Due to the depth of the restorations, a thin layer of flowable composite resin (approximately 1 mm thick) was first applied to the cavity floor and light‐cured for 20 s (Valo, Ultradent Inc., United States), followed by placement of a nanohybrid composite resin (Estelite Omega, Tokuyama, Japan). Before curing, the guide was placed over the restoration to define the correct cervical contour (Figure 6C–F). With the guide in place, excess material was carefully removed along the margins to ensure that the restoration precisely followed the virtually planned CEJ (Figure 6G,H) [14]. Light‐curing was completed with the guide in position (Valo, Ultradent Inc., United States), followed by an additional 40 s light‐exposure immediately after guide removal to ensure optimal polymerization (Figure 6I). Final contouring ensured seamless integration with the natural tooth anatomy. Finishing was performed using fine diamond burs (Komet, Germany), followed by a multi‐step polishing system with silicone polishers (Enhance Finishing and Polishing System, Dentsply Sirona, United States) and felt discs (3M Sof‐Lex Diamond Polishing System, United States) (Figure 6J,K). The restoration achieved the planned result (Figure 7).

### 2.4 Flap Preparation

**FIGURE 8:**
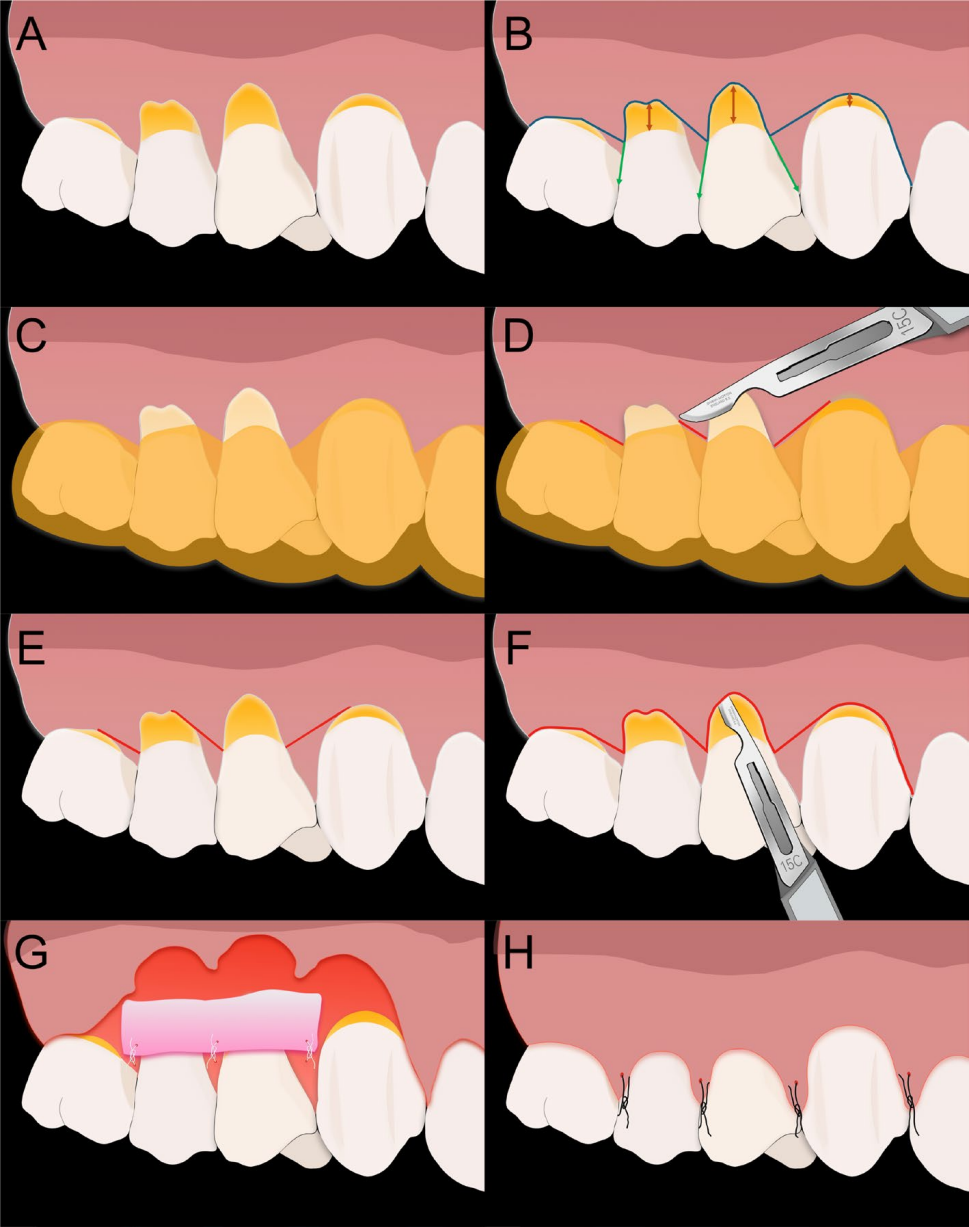
CAF surgery for root coverage using the multifunctional guide. (A) Initial aspect. (B) Planned incision pattern based on clinical parameters used for guide design. (C) Guide positioned for incision guidance. (D) Oblique incisions at the distal aspect of tooth #15 and the mesial and distal aspects of tooth #14. (E) Completion of oblique incisions at all marked points. (F) Intrasulcular incisions connecting oblique incisions, followed by tunneling at the mesial aspect of tooth #13. (G) Flap elevation and CTG suturing. (H) Final sutures.

**FIGURE 9:**
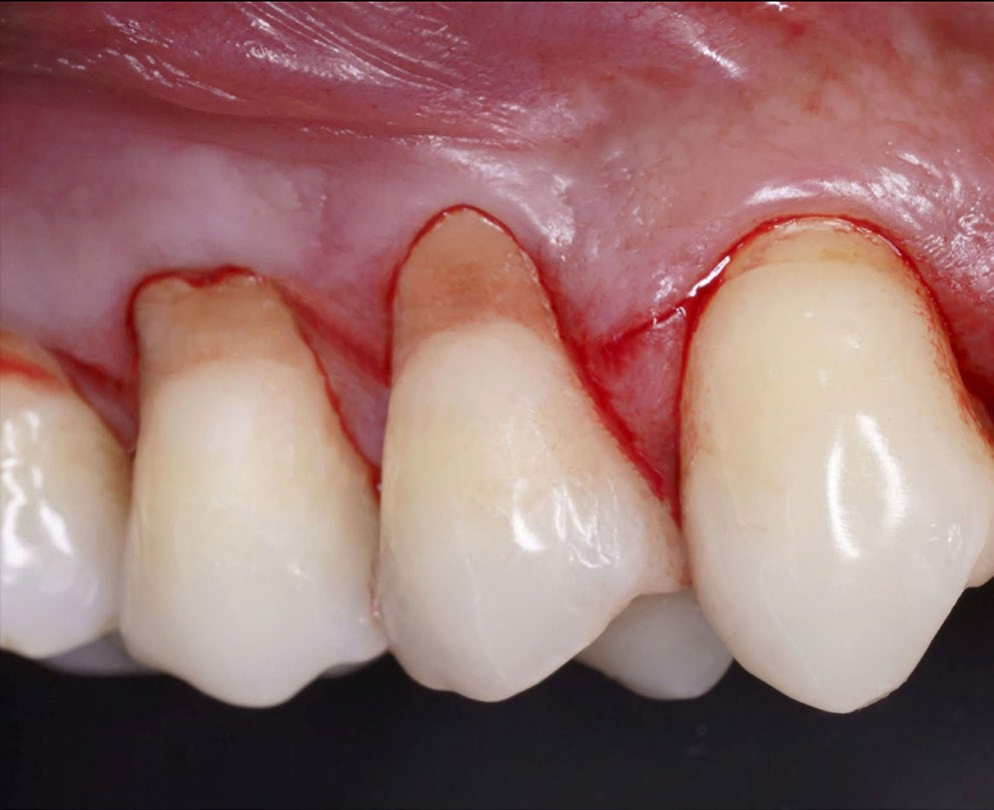
Incisions made following the virtually designed guide.

With the restorations in place, CAF surgery was performed for root coverage. Local anesthesia was administered (4% articaine and epinephrine 1:100,000). The complete sequence for performing the surgery using the multifunctional guide is shown in Figure 8. The incision pattern was guided by the previously designed surgical guide (Figure 8A,B). Oblique incisions with No. 15C scalpel blade (Carbon steel, Swann Morton, United Kingdom) were performed over the guide at the distal aspect of tooth #15 and at both the mesial and distal aspects of tooth #14 (Figure 8C–E). These were then connected by intrasulcular incisions (Figure 8F and Figure 9). A tunneling approach was performed at the mesial aspect of tooth #13. A split‐thickness flap was performed in the surgical papillae, transitioning to full‐thickness 3‐mm apical to the recession, then to split‐thickness apically. The periosteum was released to allow tension‐free flap advancement (Figure 8G).

### 2.5 Graft Harvesting and Placement

CTG was harvested from the palate using a no. 15C blade (Swann Morton, United Kingdom) with rectangular‐shaped incisions perpendicular to the bone (1.5 mm depth). The graft was de‐epithelialized extraorally [42], and the donor site was protected with sutures and a collagen sponge.

**FIGURE 10:**
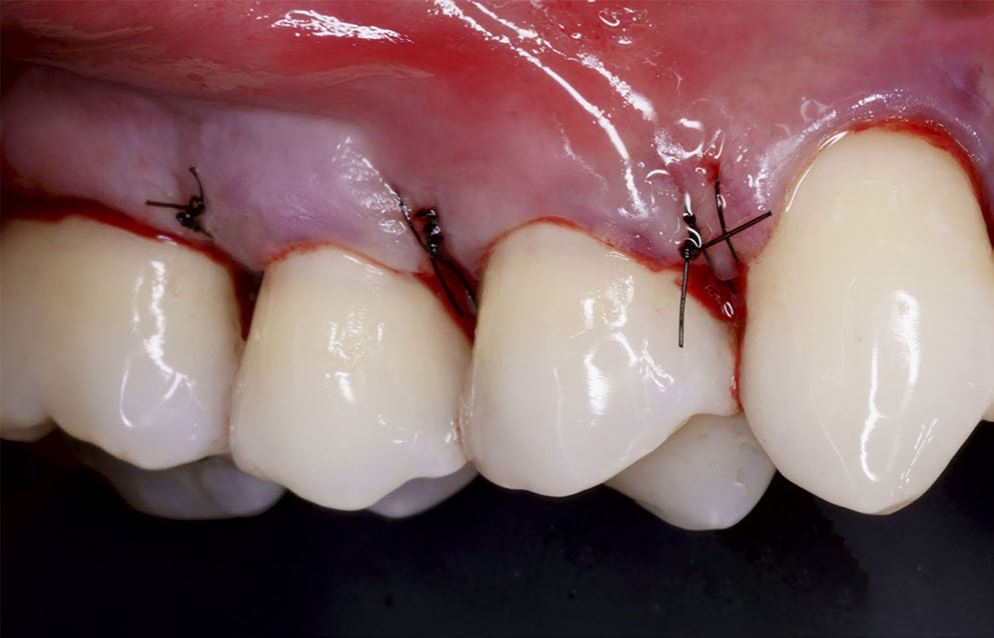
Sutures.

The graft was secured in position using simple sutures (Figure 8G). The flap was then coronally advanced to cover the previously restored CEJ by 1 mm [43] and was secured with interrupted and sling sutures (Black Monofilament Nylon Suture 6–0, Brazil) to ensure stability (Figure 8H and Figure 10).

### 2.6 Postoperative Care and Follow‐Up

Postoperative instructions included dietary restrictions, the use of 0.12% chlorhexidine rinse, and analgesics as needed. The sutures were removed 15 days postoperatively.

**FIGURE 11:**
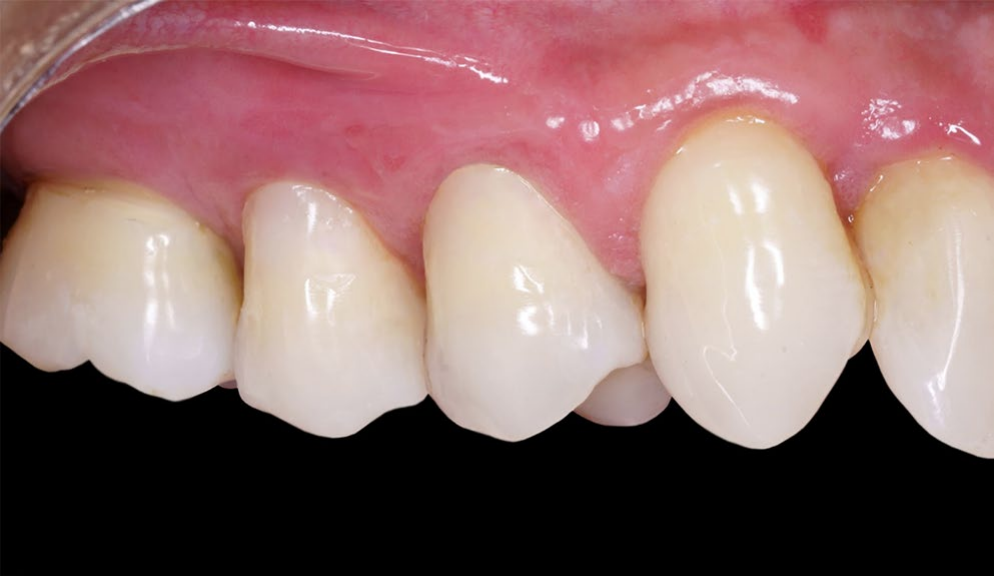
Outcome 6 months after surgery.

**FIGURE 12:**
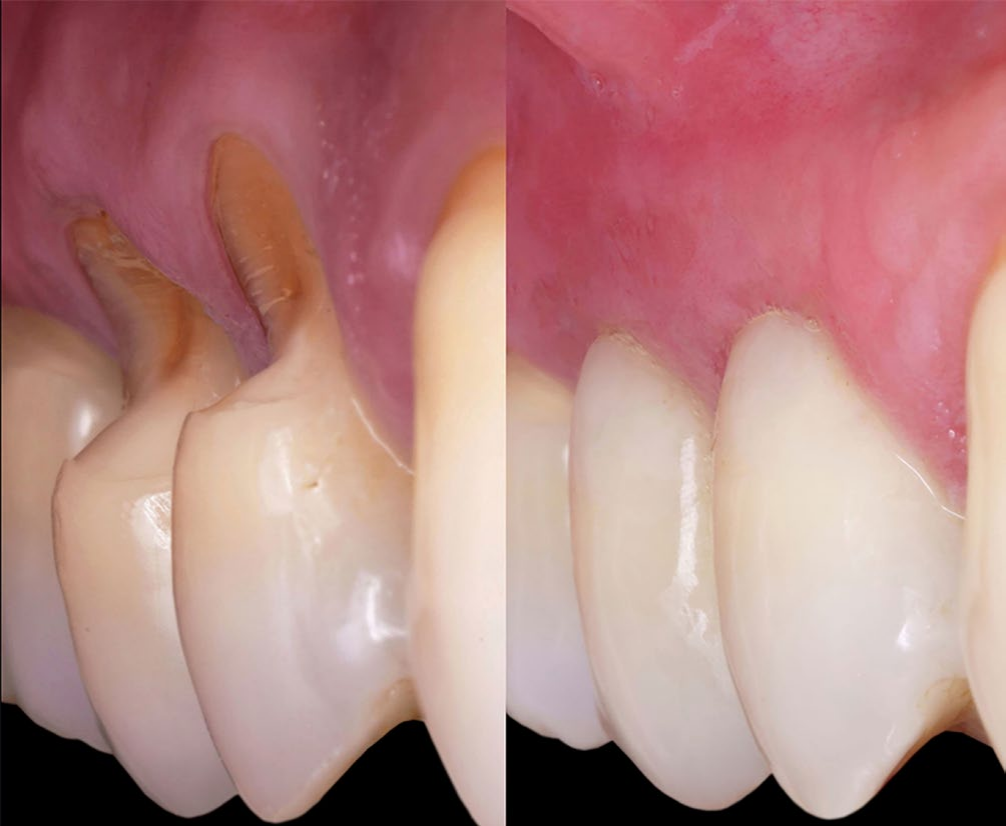
Before and after (6‐month follow‐up).

**FIGURE 13:**
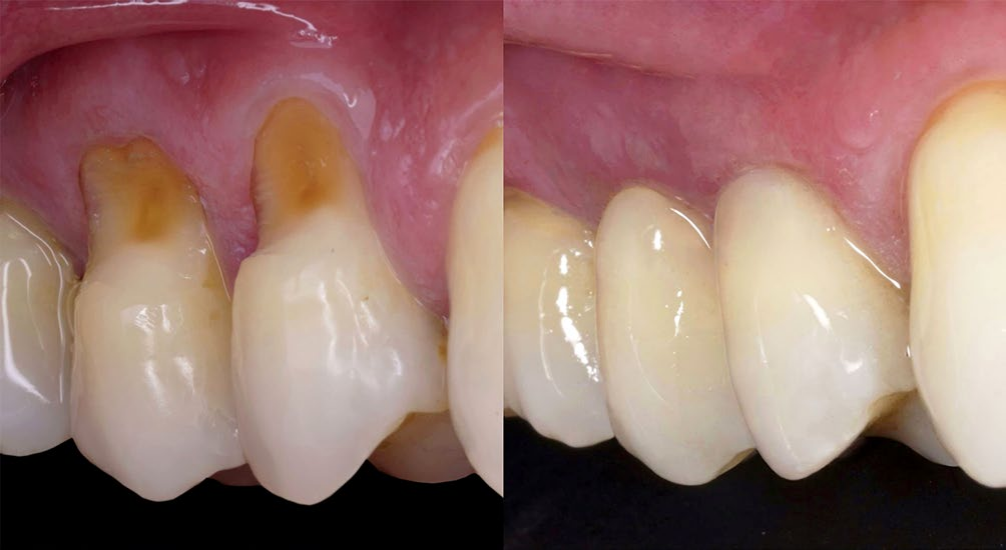
Before and after (1‐year follow‐up).

At the six‐month follow‐up, clinical evaluation confirmed a successful outcome with complete root coverage (Figures 11 and 12). This result remained stable after 1 year. The restoration also demonstrated stability, maintaining its color, polish, and surface gloss (Figure 13).

## 3 Discussion

This case report presents a digital workflow integrating restorative and surgical planning for the treatment of gingival recessions associated with NCCLs. A multifunctional guide was digitally designed and 3D‐printed to assist accurate CEJ restoration and precise surgical incisions. Following NCCL restoration, CAF combined with a CTG achieved complete root coverage. One‐year follow‐up confirmed stable gingival margins and restoration integrity.

When NCCLs are present, the CEJ is often undetectable or partially visible, making accurate estimation crucial for predictable outcomes [9, 13]. Errors in CEJ identification can compromise the surgical planning, leading to discrepancies in gingival margin adaptation. If the restoration is positioned incorrectly—leaving the coronal dentin exposed—it may lead to unintended dentin exposure after surgery, potentially being mistaken for incomplete root coverage [13, 14].

Different methods have been proposed for CEJ estimation. Zucchelli et al. suggested identifying visible CEJ portions at the mesiobuccal and distobuccal line angles and drawing an arched reference line to guide root coverage [14]. Other approaches rely on average crown lengths, proportional relationships with adjacent teeth, or homologous or contralateral teeth with intact CEJs [15]. In this case, a combination of these techniques was applied. Despite their utility, these methods can be challenging to implement during restoration; therefore, using a guide can improve accuracy and enhance treatment predictability. In addition, restoring the CEJ before surgery offers multiple advantages. It serves as a visual reference during the procedure and facilitates the establishment of a harmonized gingival margin that blends seamlessly with adjacent teeth post‐healing [13].

As for the restorative material, composite resin was used in this case due to its esthetic stability, particularly in color maintenance compared to glass ionomer cement. While both materials have shown similar outcomes in root coverage procedures [7, 13, 44] composite resin has demonstrated better marginal adaptation and esthetic longevity [13, 44]. Certain drawbacks limit the frequent use of glass ionomer cement in this context, including technical challenges due to the material's stickiness, solubility in acidic oral conditions, suboptimal esthetics, and lower bond strength to the dentin substrate, which may lead to potential retention issues [45].

In NCCLs, most of the bonded surface is dentin, with the incisal margin terminating in enamel. Therefore, bond strength to dentin is a key factor to consider when selecting the restorative material [46, 47]. Composite resins achieve superior micromechanical bonding to dentin, attributed to the establishment of a hybrid layer, consisting of demineralized collagen fibrils involved by the polymerized adhesive [45, 48]. In contrast, the bond strength of glass ionomer cements to dentin is generally inferior to that of resin composites, which may negatively impact clinical outcomes, particularly the retention of cervical restorations [45]. However, a systematic review has shown minimal differences in failure rates due to retention loss between these two materials [49]. Furthermore, it has been suggested that cervical glass ionomer cement restorations may be more prone to fractures at the cervical margin under parafunctional loads. Prior to fracture, the restorative material undergoes strain softening, compromising its structural integrity. This softening typically occurs in the cervical region, where the majority of clinical failures are observed [50]. Other factors, such as application technique, conditioner use, and interaction with bonding agents, can also influence adhesive performance [46].

Regarding the adhesive system, a 2‐step self‐etch adhesive was selected due to its favorable bonding performance in the restoration of NCCLs [46, 51]. In comparison to “total‐etch” systems, self‐etch adhesives are less sensitive to dentin's moisture conditions, virtually eliminating the risk of incomplete infiltration of the demineralized layer. Also, the presence of the monomer 10‐MDP ensures a chemical interaction between the resin and the residual mineral on the collagen fibrils, forming a stable chemical bond that makes the bonded interface less prone to degradation [51]. This chemical bonding, combined with micro‐mechanical interlocking through hybridization, accounts for the adhesive's superior bonding performance, which has been shown to result in better clinical performance, assessed by retention loss and marginal discoloration of cervical restorations, compared to other adhesive systems [49].

As for the specific composites, a flowable resin was first applied as an intermediate layer (~1 mm) [52], followed by a regular nano‐hybrid resin (~2 mm) [53]. Nano‐hybrid composite resin was chosen due to its excellent mechanical properties and polishing capabilities, showing superior smoothness results following polishing methods [54, 55]. Nanohybrid composites generally offer superior mechanical properties due to their higher filler content and optimized filler distribution [56]. Additionally, a recent in vitro study demonstrated that nanohybrid composites exhibit superior acid resistance, making them an ideal choice for patients who frequently consume acidic beverages, such as our patient [57].

A split dam technique was used for isolation, as conventional tooth‐by‐tooth isolation would have interfered with proper guide placement during the restorative procedure. While a dry field was achieved, crevicular fluid from the gingival sulcus [58] can interfere with resin adhesion in the cervical area [59], which may represent a limitation of the technique. The presence of gingival inflammation is typically associated with an increased volume of this fluid [60]. However, this risk was minimized, as the patient exhibited periodontal health and the gingival margins, due to the recessions, were positioned away from the area being restored.

Composite photo‐activation was performed using a high‐intensity light‐curing unit, according to the manufacturer's instructions. The 1‐mm flowable composite layer was light‐cured first, followed by the 2‐mm nanohybrid composite increment. The thickness of the surgical guide may have reduced the irradiance reaching the composite. For this reason, an additional light exposure was performed after the guide removal to compensate for any potential reduction in curing efficiency.

For root coverage, advanced flaps combined with a CTG remain the gold standard, offering the highest probability of achieving complete root coverage and long‐term success rates [12, 61]. Yet, the literature reveals considerable variability in both mean and complete root coverage percentages—a discrepancy that may be linked to differences in operator experience and technical execution [17, 61].

Precise incision design is critical for successful root coverage [62]. However, this step is highly operator‐dependent, and variations in execution can result in asymmetrical gingival margins and incomplete root coverage [63]. A recent clinical study demonstrated that clinicians with less experience achieved poorer outcomes with CAF surgery, characterized by reduced coverage, higher complication rates, and longer surgical times [64]. Prolonged surgical times may elevate the risk of complications due to extended tissue damage, thereby compromising healing and prognosis [65]. Conversely, employing surgical guides has been shown to mitigate adverse outcomes in procedures performed by less experienced clinicians, as evidenced in implant surgeries, while also enhancing surgical precision [66, 67].

Even experienced clinicians rely on visual judgment, which may introduce inconsistencies. A body of research indicates that surgical complications more often stem from suboptimal application of established techniques and intraoperative distractions rather than from a lack of experience or theoretical knowledge [67]. A predefined incision guide can standardize the process, minimizing subjectivity, ensuring accurate and consistent results while reducing inter‐operator variability. However, over‐reliance on guides may limit a clinician's adaptability to intraoperative variables. In other words, clinicians must balance digital assistance with intraoperative adaptability.

In addition to their common use in implant surgeries [23, 24, 25], digital planning and the use of digitally designed guides have been reported in various surgical procedures requiring precision. These include soft tissue harvesting [26], incisions for crown lengthening surgeries [30], and bone tissue cuts (e.g., designing bone windows for sinus lift procedures [27] and autogenous bone block grafts) [28]. More recently, guides have also been explored in root coverage [68]. A recently published case report described a guide designed to direct incisions in a CAF for multiple gingival recessions. On the buccal aspect, the apical limits of the guide were designed to align with and guide the intended incisions [68]. Additionally, a palatal component was included to facilitate graft harvesting with defined dimensions, as previously described in another report [26]. This palatal section was helpful for defining the margin limits for graft harvesting. By using the guide, as in our case, the incisions were performed with precision, leading to a successful outcome after 1 year [68].

The guide introduced in our report was designed for both surgical and restorative procedures in combined lesions involving NCCLs and gingival recession, achieving precise results in both stages. In these cases, obtaining a precise result in the restoration is crucial, as it also impacts the outcome of the root coverage surgery. However, transferring the restorative plan during the execution of the restoration with conventional techniques can be challenging. Although comparative studies are lacking, reports indicate that restorative procedures have also been successfully performed using virtually designed guides [31]. For instance, when digitally planned and fabricated guides were used for diastema closure using flowable composites, minimal adjustments were required due to the precision in achieving the planned result, along with reduced clinical time for performing the restorations [33, 69].

In our case, while maintaining precision, the use of the guide contributed to more time‐efficient procedures due to various factors. From a surgical perspective, at the start of a conventional CAF procedure, it is necessary to measure the length of the recessions, transfer the measurements to the interproximal area, mark the sites, and then make the incisions deliberately. In the guided approach, the guide provided all the necessary references, eliminating the need to repeat measurements already taken during planning. It also offers support for the scalpel, providing increased safety and stability during incision.

Regarding restoration, efficiency can mainly be achieved by reducing the time required for sculpting the resin to achieve the ideal shape and form. The proposed technique allowed resin application, which was then shaped by the guide and polymerized while the guide was held over the resin. However, it is important to consider that the guided approach requires clinical time dedicated to intraoral scanning and the expertise of professionals with the technology. In contrast, the conventional approach does not involve scanning or guide fabrication (designing and printing), which reduces time and costs.

Another important aspect of this approach is facilitating communication within the interdisciplinary team, compared to a traditional approach [21, 70]. The planning and design phase of the guide involved collaboration and evaluation by periodontal and restorative specialists, as well as the opportunity for all professionals involved to assess the initial condition and the plan through digital models and digital planning, with a focus on the desired outcome for both the restoration and the surgery. Additionally, the digital workflow enhanced communication with the patient by providing a clear visual representation of the treatment plan.

In this context, the guided procedure may offer both precision and time efficiency, which are not always achieved with the conventional approach, while also facilitating communication. However, aside from guided implant surgery, there is a need for comparative studies demonstrating the advantages of this guided surgical and restorative technique over conventional methods in terms of time reduction, cost‐effectiveness, precision, and accuracy. Nevertheless, all the reported cases agree that the use of digitally planned and fabricated guides has resulted in satisfactory outcomes, provided efficiency, and increased safety in performing the proposed procedures [26, 27, 28, 30, 68].

The digital workflow and guide design introduced can be adapted for different clinical cases by adjusting the restorative and surgical aspects of the guide according to the specific anatomical and clinical characteristics of each patient. A comprehensive diagnosis, which includes the evaluation of parameters related to the NCCLs and periodontal conditions, alongside the use of intraoral photographs and scanning, allows for detailed planning and assessment of the reference points for the surgery and restorative procedure. This facilitates the precise customization of both the restoration and flap design, ensuring that the guide can be tailored to various lesion sizes and depths, as well as the characteristics of gingival recession and periodontal parameters. This approach has been successfully applied in other clinical scenarios involving surgical and restorative procedures, such as crown lengthening surgeries associated with laminate veneers [71], as well as in implant surgeries and corresponding implant‐supported restorations, all tailored to the individual patient [72]. Future studies could investigate protocols for further adapting the design to accommodate a broader range of clinical variations of NCCLs and gingival recessions.

For guide design, we used Autodesk Meshmixer (Autodesk Inc., USA), a free, open‐source tool, chosen for its accessibility, suitability for our specific workflow, and the team's familiarity with the software. However, other software options could also have been used, such as Blender (Blender Foundation, Netherlands), also free and open‐source, or commercial software like Mimics (Materialize NV, Belgium), designed specifically for medical purposes, as well as Exocad (Exocad GmbH, Germany), Nemotec (Nemotec S.L., Spain), both specifically designed for dental applications. Each of these tools has its own set of features and applications. It is important to note that while the design results may be similar, these tools can differ significantly in terms of usability, cost, and efficiency, largely depending on the operator's familiarity and ability [73].

Regarding the digital workflow, the design and fabrication of the guide were carried out in an external laboratory, while the clinical hours in the office were exclusively dedicated to patient care (clinical examination, surgical and restorative procedures, and follow‐up appointments). However, a chairside workflow, involving the design and fabrication of the guide within the dental office, could also be a viable option [74, 75], if the practitioner has the necessary expertise, software, and equipment.

Finally, although the results of this case report are promising, reports with extended follow‐up periods are needed to confirm the stability of both the restoration [76] and the soft tissue [12], and to assess the long‐term durability and predictability of the proposed treatment approach. Further research should also explore how digital guides can be refined for broader applications in NCCLs and gingival recession management.

## 4 Conclusion

This case report highlights the benefits of a 3D‐printed multifunctional digital guide for precise CEJ restoration and flap design in treating gingival recessions associated with NCCLs. One‐year follow‐up demonstrated stable complete root coverage and restoration success in terms of marginal adaptation, color stability, and surface integrity.

## Conflicts of Interest

The authors declare no conflicts of interest.

## Data Availability

The data that support the findings of this study are available from the corresponding author upon reasonable request.
